# Dutasteride plus Tamsulosin fixed-dose combination first-line therapy versus Tamsulosin Monotherapy in the treatment of benign prostatic hyperplasia: a budget impact analysis in the Greek healthcare setting

**DOI:** 10.1186/1471-2490-14-78

**Published:** 2014-09-26

**Authors:** Maria Geitona, Pinelopi Karabela, Ioannis A Katsoulis, Hara Kousoulakou, Eleni Lyberopoulou, Eleftherios Bitros, Loukas Xaplanteris, Sotiria Papanicolaou

**Affiliations:** 1 School of Social Sciences, University of Peloponnese, Corinth, Greece; 2 GlaxoSmithKline, Athens, Greece; 3 PRMA consulting, Athens, Greece

**Keywords:** Benign prostate hyperplasia, Dutasteride plus tamsulosin fixed-dose combination, Budget impact, Costs, Health resources

## Abstract

**Background:**

The purpose of this study was to explore the budget impact of dutasteride plus tamsulosin fixed-dose combination (DUT + TAM FDC) versus tamsulosin monotherapy, in the treatment of patients with benign prostatic hyperplasia (BPH) from the perspective of the Greek healthcare insurance system.

**Methods:**

A Microsoft Excel-based model was developed to estimate the financial consequences of adopting DUT + TAM FDC within the Greek healthcare setting. The model, compared six mutually exclusive health states in two alternative treatment options: current standard of care and the introduction of DUT + TAM FDC in the market. The model used clinical inputs from the CombAT study; data on resource use associated with the management of BPH in Greece were derived from expert panel, and unit cost data were derived from official reimbursement tariffs. A payer perspective was taken into account. As patient distribution data between public and private sectors are not available in Greece two scenarios were investigated, considering the whole eligible population in each scenario. A 4 year time horizon was taken into account and included treatment costs, number of transurethral resections of the prostate (TURPs) and acute urinary retention (AUR) episodes avoided.

**Results:**

The clinical benefit from the market adoption of DUT + TAM FDC in Greece was 1,758 TURPs and 972 episodes of AUR avoided cumulatively in a four year period. The increase in total costs from the gradual introduction of DUT + TAM FDC to the Greek healthcare system ranges from €1.3 million in the first year to €5.8 million in the fourth year, for the public sector, and €1.2 million to €4.0 million, for the private sector. This represents an increase of 1.91% to 7.94% for the public sector and 1.10% 3.29% in the private sector, during the 4-year time horizon.

**Conclusions:**

Budget impact analysis (BIA) results indicated that the gradual introduction of DUT + TAM FDC, would increase the overall budget of the disease, however providing better clinical outcomes. DUT + TAM FDC drug acquisition cost is partly offset by the reduction in the costs associated with the treatment of the disease.

## Background

Benign prostatic hyperplasia (BPH), a common benign neoplasm in men, is a chronic condition with an age dependent epidemiology. It is associated with progressive lower urinary tract symptoms (LUTS) and affects 75% of men older than 70 [1]. Although many epidemiological clinical studies have been conducted worldwide over the last 20 years, the prevalence of clinical BPH remains difficult to determine. A broadly accepted clinical definition of BPH is lacking, and thus performance of adequate epidemiological studies is hampered [2]. A commonly occurring condition in men with underlying BPH is acute urinary retention (AUR). AUR is an uncomfortable and potentially life-threatening condition characterized by a sudden inability to urinate associated with intense suprapubic discomfort. Medical intervention is often required in order to relieve the severe discomfort experienced by patients with AUR [3]. Overall, the common clinical manifestations attributed to BPH include LUTS, urinary tract infection, incomplete bladder emptying, acute and chronic urinary retention, chronic renal insufficiency, urosepsis, and hematuria [1].

Therapeutic interventions for LUTS, due to BPH, provide sustained improvement in clinical symptoms and quality of life (QoL), while inhibiting progression of the condition [4]. The two main pharmacological agents for the management of BPH/LUTS are 5-alpha-reductase inhibitors (5-ARIs) and alpha-blockers. Dutasteride is a 5-ARI and works by blocking the conversion of testosterone to dihydrotestosterone, thus reducing cellular growth and in turn reducing the size of the prostate [5]. Tamsulosin is an uroselective alpha-blocker and exerts its activity by relaxing bladder neck muscles and prostate muscle fibres that in turn improve in urine flow rate [6]. Combination therapy was significantly superior to both monotherapies at reducing the relative risk of BPH clinical progression, as concluded by the Combination of Avodart^TM^ (dutasteride) and Tamsulosin (CombAT) study. CombAT was a randomised, multicentre, double-blind, parallel-group study in 4,844 men of 50 years or older with a clinical diagnosis of BPH for the treatment of moderate to severe BPH that spanned over 4 years [7].

The increasing use of pharmacological agents, during the past twenty years, has transformed the management of BPH as shown by a dramatic decrease in the use of transurethral prostatectomy (TURPs), inpatient hospitalization, length of hospital stay and an increase in the number of outpatient visits for the condition, in the US [8]. In 2000, the direct cost of BPH treatment in the US was estimated to be US$1.1 billion exclusive of outpatient pharmaceuticals [8]. Another study conducted in UK estimated the annual economic burden of BPH ranged between £62 million and £91 million, excluding the intangible costs [9].

Recent economic evaluations have been undertaken with a specific focus on pharmaceutical intervention related to treatment of BPH. Specifically for the DUT + TAM FDC vs. tamsulosin monotherapy, economic analyses have been conducted in the UK [10], Canada [11], Spain [12], and Scandinavia [13], where the fixed-dose combination therapy was shown to be cost-effective compared to tamsulosin monotherapy.

The aim of this study was to assess the budget impact of the fixed dose combination dutasteride and tamsulosin (DUT + TAM FDC) versus tamsulosin monotherapy for the treatment of moderate to severe BPH in Greece.

## Methods

A budget impact analysis was conducted based on a Markov decision model for the treatment of moderate to severe BPH comparing DUT + TAM FDC combination therapy over tamsulosin monotherapy. The model was populated with local healthcare resource utilisation estimates, unit costs and epidemiological data. Clinical efficacy data was retrieved from the ComBAT study. Univariate sensitivity was conducted by examining changes in the prevalence of BPH, number of patients based on prostate volume, and success rate of TURPs.

### Model description

**Figure 1:**
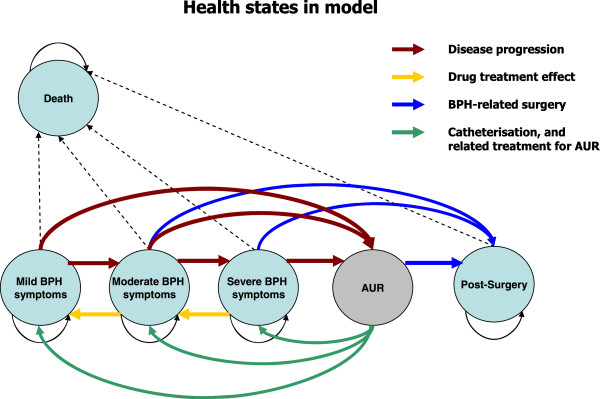
Markov model structure and health states.

The pharmacoeconomic analysis was conducted based on a Markov decision model developed with Microsoft Excel. Using data from the CombAT study [7] the analysis was based on a Markov stochastic process with 6 mutually exclusive health states iterated over 3 month cycles for a total of 4 years (Figure 1 Markov model structure and health states). A Markov model is a decision analytic technique that allows simulation of disease progression during a defined period of time, and is particularly suitable to model medical conditions that involve uncertainty over a long time horizon and/or recurrent events [14].

The six discrete health states (or ‘Markov states’) which simulated each possible clinical event and described disease progression, were the following: ‘mild’, ‘moderate’, ‘severe’ BPH, based on IPSS symptom severity as defined in the CombAT trial, AUR, post-surgery and death [10]. The AUR was modelled as a temporary health state. Successful treatment of AUR with emergency catheterization or trial without catheter (TWOC) would return the patients to the previous health state.

Unsuccessful treatment would lead to the post-surgery health state with the implication of a BPH-related surgery.

The model characteristics have been based on the Walker et al. study [10]. Although there are a number of surgical options for BPH, the model assumed all patients have TURP when surgery is indicated. The use of TURP has been the gold standard for decades [15] and the American Urological Association guidelines consider this intervention as the benchmark for surgical therapies [16], while the European Urological Association guidelines report TURP as the preferred treatment for prostate sizes ranging from 30 to 80 mL [2]. Patients undergoing TURP enter the post-surgery health state, where they remain until the end of the 4-year time horizon or death. A patient can undergo up to two TURP procedures (after failure of the first procedure or relapse).

Assumptions regarding the progression of the condition over time and BPH events, such as AUR, surgery or death had to be made for the model. Transition probabilities between health states [10] were derived directly from the CombAT study individual patient-level data [7] and the clinical study report. Patient-level data related to the mild, moderate and severe ‘Markov’ health states were available from follow-up visits every 3 months and accordingly probabilities for transitions between each of these health states were derived. For AUR and post-surgery health states, the three-month transition probabilities were calculated from the number of yearly events using standard methods as described by Briggs *et al*. [17]. For patients who experienced AUR, the care pathway was not reported in the CombAT trial. thus, the model assumed that 50% of TWOC procedures are successful based on a clinical review by Emberton *et al.*[18]. Furthermore, patients who underwent BPH-related surgery followed the care pathway that is published in international literature [19].

The European Urology Association BPH treatment guidelines helped inform the probability of any adverse event associated with TURP [2]. This total probability was applied to all patients in the post-surgery state, regardless of the success or failure of the procedure. Adverse events (AEs) associated with medical therapy were based on the CombAT trial. However, since the percentage of patients experiencing serious drug-related AEs was <1% in all treatment arms of the CombAT trial, these were excluded from the analysis.

Overall, the model simulated and compared:

• Standard of Care (SoC): Patients are treated with tamsulosin only, representing current standard care.

• DUT + TAM FDC: Gradual introduction of DUT + TAM FDC therapy in the treatment of BPH, with defined market share gains over time.

In each of the treatments compared, the health costs allocated to each discrete state were accumulated, through the 4-year time horizon [10].

**Figure 2:**
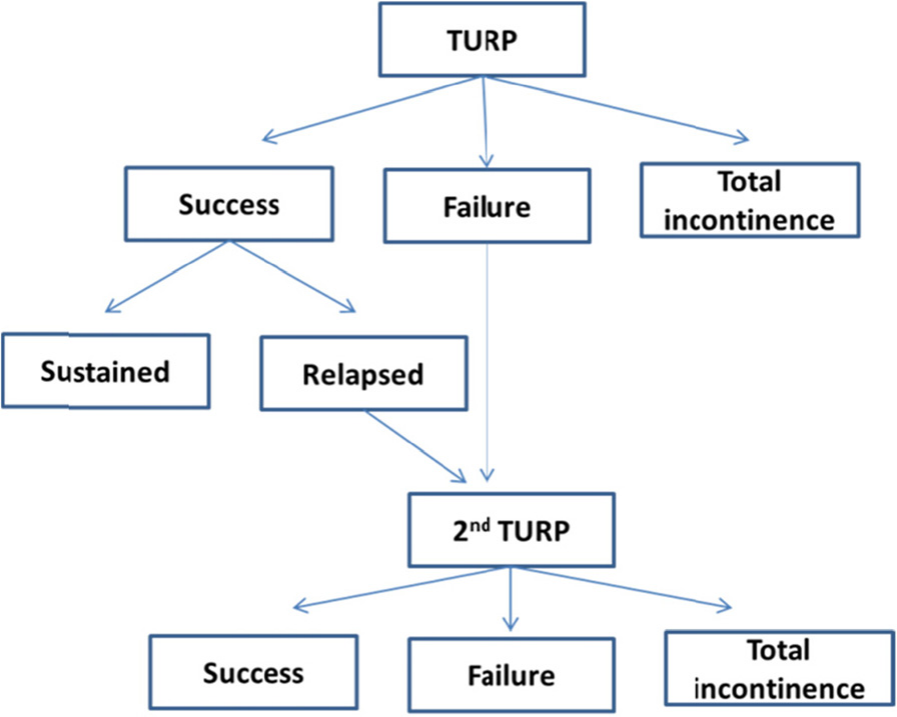
Post surgery pathway.

The Markov model was designed assuming that treatment switching only occurred at the end of each year and that patients remained on DUT + TAM FDC therapy upon regression to mild BPH symptoms. This is a plausible clinical assumption as according to a study from Toren *et al*. [20], preventive administration of 5-ARI could decrease the incidence of BPH clinical progression, which was validated during the expert panel. The model assumed 100% compliance with pharmacotherapy and that patients incur different resource use and costs according to the severity of their symptoms. The patients who entered the model had an initial urology consultation at a higher cost. The costs described in Table 1 only applied to patients entering the model from year two onwards, since patients in the model in year one were assumed to have already had their initial urologist consultation. The AUR ‘Markov’ state was modelled as a tunnel state which occurred at mid-cycle length and no patients were assumed to be in the AUR state at the beginning of each cycle. Non AUR patients were only assumed to undergo TURP procedures from the ‘moderate’ and ‘severe’ symptom severity health states. No patients were assumed to die while undergoing treatment for AUR; all-cause mortality was applied to patients at the beginning of each cycle. Patients who developed AUR, following immediate catheterization, underwent TWOC. If this was successful, they returned to their previous symptom severity ‘Markov’ state. The AUR state was modelled in this way to reflect that successful catheterization and TWOC had no effect on disease progression.The post-surgery ‘Markov’ state was modeled according to Figure 2 Post surgery pathway, with the assumption that patients underwent only one type of BPH surgery, a TURP.Furthermore, patients were assumed to undergo a TURP procedure when the TWOC procedure failed. Patients undergoing a TURP procedure entered a ‘Post surgery’ health state and followed the pathway shown in Figure 2. In this decision diagram ‘failure’ was defined when patients did not achieve >50% reduction in the IPSS score after surgery.

**Table 1:** Unit costs by healthcare sector

**Exam/laboratory test**	**Scenario**	**Unit cost (€)**	**Reference**
*Consultation*			
**Cost per follow up urologist visit**	Public	10.00	National Organization for the Provision of Healthcare Services (EOPYY) ( http://www.eopyy.gov.gr) accessed 1 October 2012
	Private	50.00	Average price confirmed by expert panel consensus.
**Cost per serum creatinine test**	Public	4.05	Social Security Institution IKA* tariff (PD157/55)
	Private	16.00	Biomedicine SA price (provided 1 October 2012)**
**Cost per urodynamic test**	Public	18.99	Social Security Institution IKA tariff (PD157/55)
	Private	268.50	Average price from pricelists of two major private hospitals in Athens (‘Hygeia’ hospital and ‘Iaso’ general hospital)
**Cost per flexible cystoscopy**	Public	4.05	Social Security Institution IKA tariff column A (PD 15766 surgical)
	Private	650.00	Average price from pricelists of two major private hospitals in Athens (Hygeia hospital and Iatriko Athinon hospital)
*Procedure*			
**Cost of prostate related surgery without complications**	Public	1,007.00	DRG list FEK 946 -12Mar2012 (DRG B02Χ)
	Private	1,000.00	DRG list FEK 946 -12Mar2012 (DRG Y05Χ)
**Cost of prostate related surgery with complications**	Public	2,127.00	Average of DRGs Y05M (cost of prostate related surgery with complications) and B02M (cost of prostate related surgery with complications) (FEK 946 -12Mar2012).
	Private	2,848.00	DRG Y05M (FEK 946–12 Mar2012)
**Cost per episode of AUR (non-elective)**	Public	7.63	Social Security Institution IKA tariff for catheterization (PD 157/3, 157/65 surgical)
	Private	50.00	Average price confirmed by expert panel consensus

*IKA Social Security fund.

**Biomedicine SA is a leading primary healthcare services provider in Greece.

### Model inputs

#### Patient population

A cohort of Greek male patients aged ≥50 years, diagnosed with moderate to severe BPH, as defined by an International Prostate Symptom Score (IPSS) populated the model in order to resemble the population characteristics of the CombAT study. Due to lack of national epidemiological data on BPH, the internationally accepted prevalence of BPH in the aforementioned age group was used and set at 30%, while the proportion of patients diagnosed with moderate to severe BPH was 63%, as documented in international and European studies [21-23]. Based on the 2001 Greek population census, the men population aged over 50 was 1,724,867 and consequently, the estimated number of men entering the model was set at 326,000 (=1,724,867*0,3*0,63).

The initial distribution of patients in each of the BPH symptom health states that have entered the model was defined by the baseline IPSS of the CombAT trial. Specifically, 7% of patients had improved to mild symptoms (IPSS <12) before starting treatment and the remaining 93% of patients would be considered as moderate to severe condition (63% and 30% moderate and severe, respectively) [11]. The IPSS range for mild symptoms is 0–7, moderate 8–19 and severe 20–35. The model allowed new patients to enter based on BPH incidence in accordance with the International Society of Pharmacoeconomics and Outcomes Research (ISPOR) guidelines [24].

### Clinical data

The CombAT trial was a 4-year randomized double-blind parallel group study in 4,844 men ≥50 yrs. of age with clinically diagnosed moderate to severe BPH, IPSS ≥12, prostate volume ≥30 ml, and serum prostate specific antigen (PSA) 1.5–10 ng/ml [7].

The most common aetiology of male LUTS is BPH, thus the incidence of BPH was estimated from the incident of recorded male LUTS in the Netherlands, according to the Verhamme *et al.* study [23]. Based on this study, the calculated incidence in men aged older than 50 years (2,090) was divided by the number of man-years (110,321) equalling 18.94 per 1,000 man-years.

For the overall mortality weighing, the mortality rates were calculated from the World Health Organization (WHO) health statistics and health information system interim life tables [25]. The annual risk of death in 2009 was extracted for Greek men aged >50 years.

### Resource utilization data

The medical resource use regarding the management of patients with BPH in Greece was not available from existing literature or valid national data sources, thus this information was retrieved from a local expert panel and use of the Delphi technique [26]. A panel of 9 clinical experts in urology was assembled in order to collect primary data regarding patient management patterns for BPH in the local healthcare setting. The synthesis of the panel was geographically representative and consisted of urologists from academic institutions and major city hospitals in Greece. The questions related to the medical resource use were projected on a screen and the expert panel was asked to enter their estimates using a handheld tele-voting system. Consensus was achieved using the Delphi method with up to two iterations of open discussion followed by re-voting from the panellists. The average of the experts’ answers was then included in the model grouped by routine care of patients with BPH, treatment of AUR and TURP consultations.

No Ethics Committee approval was requested for the primary research component of the study, as the conduct of interviews with physicians and experts’ panels are not subject to any approval according to the Greek legislation.

### Cost data

Costs used in the model are in nominal 2013 Euros and were not discounted nor inflated, as recommended by international guidelines for BIA [24].

Drug costs for tamsulosin and DUT + TAM FDC were based on the retail prices (Price Bulletin 15 February 2013, ΥΥKA 2013/2). Specifically DUT + TAM FDC was priced at €30.02 and tamsulosin at €12,42 (which is the volume-weighted average price of originator and generics according to market share data provided by IMS Health Greece ( http://www.imshealth.com).

As serious drug-related AEs were <1%,% in all treatment arms of the CombAT trial, these were excluded from the analysis.

Costs of consultations, procedures and laboratory tests were taken from officially published public tariffs and private hospitals in Athens. Hospitalization costs were based on the DRG list from the Greek Ministry of Health, (official government gazette March 2012 [27]).

The unit costs for the private and public sector setting are presented in Table 1.

### Market data

DUT + TAM FDC has been available in Greece since 2011, thus market share data (provided by IMS Health Greece) were used as input for the model for the first two and a half years of the analysis.

Market uptake of DUT + TAM FDC was defined as the percentage of the total first-line treatment BPH market that is gained every year. In particular, DUT + TAM FDC gained 2.0%, 4.0%, 4.5% and 11.0% of market share in the 4-years’ time horizon used in the model. Moreover, the percent of moderate to severe BPH patients, who switched from SoC to DUT + TAM FDC, at the end of every year, was set to 3.0%, 4.0%, and 5.5%.

### Perspective of analysis

The study was conducted from the payer perspective, and in particular two different scenarios were investigated: the public sector scenario, which includes the costs reimbursed by social insurance funds, and the private sector scenario, which includes the costs incurred by patients and private health insurance. Because there are no published estimates of the percentages of the respective sectors in the total healthcare setting, these two were explored separately as two extreme scenarios.

### Model outputs

The model estimated the following outputs thought the 4-year time horizon from the introduction of DUT + TAM FDC in the Greek market. Clinical outcomes refer to the number of TURPS or number of episodes of AUR avoided as well as the incremental cost per AUR avoided. Economic outputs include total costs (i.e., costs of drug treatment, costs of consultations, costs of AUR and costs of BPH related surgery).

## Results

### Resource utilization

Table 2 shows the resource utilisation of BPH patients, which the expert panel affirmed was consistent across both the social and private healthcare setting. Patients are treated and followed up directly by urologists for all prostate related complication, while visits to GPs are limited to remote areas.

**Table 2:** Routine care for patients with BPH

**Resource use**	**Average results**
**Patients with ‘Mild’ IPSS score**	
Number of GP visits in first year	1.22
Number of GP visits per year	0.44
Number of urologist visits per year in subsequent years	1.11
**Patients with ‘Moderate’ IPSS score**	
Number of urologist visits in first year	2.22
Number of GP visits per year	2
Number of urologist visits per year in subsequent years	2.39
**Patients with ‘Severe’ IPSS score**	
Number of urologist visits in first year	2.56
Number of GP visits per year	0
Number of urologist visits per year in subsequent years	2.78
Number of flexible cystoscopies per year	0.39
Proportion of patients undergoing flexible cystoscopy	7%
**Successful TURP**	
Number of peri-operative urologist consultations	2.67
Number of follow up urodynamic tests	0.06
**Unsuccessful TURP**	
Number of peri-operative urologist consultations	3.89
Number of follow up urodynamic tests	0.06
**Patients with total and permanent incontinence**	
Number of urologist visits per year	4.56
**Procedures with complications**	
Number of follow up urologist visits	3.44
**All procedures**	
Number of peri-operative flexible cystoscopies	1.1

Abbreviations: *IPSS* International Prostate Symptom Score, *GP* general practitioner.

### Model results

#### Clinical outcomes

Table 3 presents the clinical outcomes of the gradual introduction of DUT + TAM FDC, in the 4-year time horizon of the model.

**Table 3:** Clinical results

**Year**	**Tamsulosin monotherapy (SoC)**		**Tamsulosin monotherapy & gradual introduction of DUT + TAM FDC**		**Number avoided**	
	**TURP**	**AUR episodes**	**TURP**	**AUR episodes**	**TURPS**	**AUR episodes**
**1**	7,145	5,786	7,102	5,726	42	60
**2**	12,507	6,366	12,147	6,198	360	168
**3**	9,637	5,943	9,215	5,702	422	241
**4**	11,475	7,183	10,541	6,679	934	503
Total	**40,763**	**25,277**	**39,006**	**24,305**	**1,758**	**942**

### Cost outputs

#### Public sector scenario

Table 4, presents the total economic impact from the gradual introduction of DUT + TAM FDC to the various cost components of BPH disease managment (i.e., consultations, surgery, AUR costs, drug costs) in the public sector.

**Table 4:** Cost analysis of public sector

**SoC: Tamsulosin monotherapy**						**Total budget impact (%)**
**Year**	**Consultation costs (€)**	**Surgery costs (€)**	**AUR costs (€)**	**Drug costs (€)**	**Total costs (€)**	
**1**	11,497,557	8,628,675	44,144	47,577,203	67,747,579	-
**2**	11,527,061	15,104,305	48,573	47,785,803	74,465,743	**-**
**3**	11,260,338	11,638,737	45,344	47,734,616	70,679,036	**-**
**4**	11,412,776	13,858,461	54,803	47,798,059	73,124,100	**-**
Tamsulosin monotherapy & gradual introduction of DUT + TAM FDC						
**1**	11,492,585	8,577,613	43,687	48,930,196	69,044,082	-
**2**	11,404,330	14,669,865	47,289	50,606,379	76,727,862	**-**
**3**	11,102,755	11,128,827	43,506	52,334,084	74,609,173	**-**
**4**	11,173,679	12,730,920	50,963	54,976,669	78,932,231	**-**
Budget impact						
**1**	-4,972	-51,061	-457	1,352,994	1,296,503	1.91
**2**	-122,731	-434,440	-1,284	2,820,575	2,262,120	3.04
**3**	-157,583	-509,909	-1,838	4,599,467	3,930,137	5.56
**4**	-239,097	-1,127,541	-3,840	7,178,610	5,808,131	7.94

#### Private sector scenario

Table 5 presents the total economic impact of gradual introduction of DUT + TAM FDC to the various cost components of BPH disease managment (i.e., consultations, surgery, AUR costs, drug costs) in the private sector.

**Table 5:** Cost analysis of private sector

**Year**	**Consultation costs (€)**	**Surgery costs (€)**	**AUR costs (€)**	**Drug costs (€)**	**Total costs (€)**	**Total budget impact (%)**
SoC: Tamsulosin monotherapy						
**1**	49,924,365	14,706,649	289,282	47,577,203	112,497,498	-
**2**	50,090,297	25,743,665	318,303	47,785,803	123,938,068	-
**3**	48,962,652	19,836,976	297,142	47,734,616	116,831,388	-
**4**	49,630,480	23,620,258	359,129	47,798,059	121,407,926	-
Tamsulosin monotherapy & gradual introduction of DUT + TAM FDC						
**1**	49,898,950	14,619,620	286,287	48,930,196	113,735,054	-
**2**	49,458,768	25,003,208	309,888	50,606,379	125,378,243	-
**3**	48,142,186	18,967,890	285,101	52,334,084	119,729,260	-
**4**	48,397,466	21,698,485	333,965	54,976,669	125,406,586	-
Budget impact						
**1**	-25,415	-87,028	-2,995	1,352,994	1,237,555	1.10
**2**	-631,529	-740,457	-8,415	2,820,575	1,440,175	1.16
**3**	-820,467	-869,086	-12,042	4,599,467	2,897,873	2.48
**4**	-1,233,014	-1,921,773	-25,163	7,178,610	3,998,659	3.29

### Sensitivity analysis

In order to assess the impact of uncertainty of various model inputs on the results of the study, univariate sensitivity analyses were conducted on two variables that contributed to the cost of treatment: eligible patient population and higher success rates of TURPs.

The prevalence of BPH was allowed to vary by ±10% in one-way sensitivity analyses, in order to reflect the uncertainty due to lack of local epidemiological data (Table 6).

**Table 6:** Sensitivity analysis of net budget impact of DUT + TAM FDC (€)

**Year**	**1**	**2**	**3**	**4**
**Public sector**	1,296,503	2,262,120	3,930,137	5,808,131
**Prevalence of BPH**				
20%	864,336	1,553,230	2,752,483	4,195,474
40%	1,728,671	2,971,017	5,107,791	7,420,837
**Only BPV > 50 cc**	537,096	1,142,338	1,886,053	2,934,517
**Probability that first and second TURPs are successful**				
88%	1,300,878	2,299,356	3,973,923	5,904,891
99%	1,305,243	2,336,509	4,017,601	6,001,441
**Private sector**	1,237,555	1,440,175	2,897,873	3,998,659
**Prevalence of BPH**				
20%	825,037	993,147	2,031,237	2,892,582
40%	1,650,074	1,887,239	3,764,509	5,104,980
**Only BPV > 50 cc**	422,530	719,264	1,263,342	2,003,231
**Probability that first and second TURPs are successful**				
88%	1,245,058	1,504,091	2,973,305	4,165,148
99%	1,252,516	1,567,598	3,048,198	4,330,599

A separate analysis estimated the budget impact from the introduction of DUT + TAM FDC when the eligible population included only patients with a baseline prostate volume > 50 mL (Table 6) which according to clinical practice is viewed as enlarged.

Additionally the success rate of surgical procedures in terms of symptoms improvement was allowed to vary by +11% and +22% in order to present the variation of clinical practice [28-30]. These adjustments are aligned with the assumption that no surgical re-treatment would be required after initial operation.

Sensitivity analysis results indicate that the overall budget for the treatment of BPH increases with use of DUT + TAM FDC compared to tamsulosin monotherapy during the 4 year study period (Table 6), for either of the two scenarios (private and public sector).

## Discussion

The present study is the first to estimate the budget impact from the introduction of DUT + TAM FDC therapy in the treatment of moderate to severe BPH, in the Greek health care setting. The study estimated that the gradual introduction of DUT + TAM FDC would result in avoidance of 1,758 TURPs and 972 AURs, in total over the 4-year time horizon, compared with tamsulosin monotherapy. In terms of BIA, the study showed that the introduction of the new therapeutic intervention would increase the disease management budget by 1.9% in year one, up to 7.9% in year four from the public sector perspective, and by 1.1% to 3.3% respectively from the private sector perspective. The observed increase is driven from pharmaceutical costs which are partly offset by the reduction in the costs associated with the overall treatment of the disease. In particular, savings associated with the use of combination therapy arise from the reduction in consultations, surgeries and AURs. These savings are estimated at €1.13 and €1.95 million in the public and private sector scenarios, respectively.

The biggest challenge in the present study was to estimate model inputs, due to lack of local data in published literature. Clinical data were based on the pivotal trial (the ComBAT study), incidence, prevalence of BPH and LUTS [29] data were extracted from European sources [22,23], and were subsequently confirmed by the expert panel. Our analysis was performed with inclusion of all patients, regardless of pre-treatment prostate volume (326,000 men estimation). Resource utilisation data were collected via the expert panel with the use of the Delphi technique, while local unit cost data were collected through publicly available sources.

Among the parameters examined in the sensitivity analyses, prevalence data had the highest impact in the results, due to the change of eligible patients number that enter the model.

There are certain limitations in the current study. The analysis was performed with a computer based model, which raised the need for adoption of simplifications and assumptions that may have not reflected real life data and may have created uncertainties. However, these uncertainties were minimized through the use of expert opinion. Due to lack of strong supporting evidence, such as registries or officially published sources, expert opinions were used to gather data regarding health resource utilization.

In addition, the lack of data on the patient distribution between the public and private sector in Greece has not allowed the generation of a weighted average for the total costs. Therefore, to overcome this limitation two extreme scenarios of public and private sectors and the relevant unit costs, were estimated separately. The differences observed between the two sectors’ unit cost data are due to the fact that public sector reimbursement rates do not reflect real cost, as they have not been updated since 1992 [31]. The two scenarios aimed at providing a range within which true costs lie.

Our findings may be used to inform the development of health policy resource allocation decisions regarding the pharmaceutical treatment of BPH. However, further research and additional empirical data are necessary in order to estimate more accurately the cost of DUT + TAM FDC, and also incorporate quality of life estimates that these patients experience compared to the currently administered monotherapies, in the Greek healthcare setting. Especially in the environment of economic recession that Greece is experiencing, economic evaluation studies that would reveal the value-for-money of new therapies can help policy decision makers rely on evidence based treatments and achieve health insurance fund sustainability.

## Conclusions

BIA results indicated that the gradual introduction of DUT + TAM FDC, increases the overall budget of the disease while providing better clinical outcomes in terms of avoidance of 1,758 TURPs and 972 AUR episodes. These results can be used in the decision-making process for resource allocation purposes. Further research on patient reported outcomes and additional economic evaluation studies that would incorporate the incremental monetary effect with the quality-adjusted life year that BPH patients gain from DUT + TAM FDC, compared to the currently administered monotherapies, would reveal the value-for-money estimate of the fixed-dose combination therapy in the Greek healthcare system.

## Competing interests

Funding for the research and preparation of this article was provided by GlaxoSmithKline, which approved the study design and manuscript. At the time of the analysis, Pinelopi Karabela, Eleni Lyberopoulou, Eleftherios Bitros and Loukas Xaplanteris were full- time employees of GlaxoSmithKline. Hara Kousoulakou, Ioannis Katsoulis, and Sotiria Papanicolaou were employees of PRMA Consulting, a consultant to GlaxoSmithKline.

## Authors’ contributions

PK, HK, EL, LX and SP participated in the design and coordination of the study and the analysis and interpretation of the data, and drafted the manuscript. EB revised the manuscript critically for important intellectual content. MG, PK, IAK, HK, LX and SP participated in the expert panel for the validation of the data on medical resource utilization and contributed to the manuscript preparation. All authors read and approved the final manuscript.

## Pre-publication history

The pre-publication history for this paper can be accessed here:

http://www.biomedcentral.com/1471-2490/14/78/prepub
